# Editorial: Expert opinions in genitourinary oncology

**DOI:** 10.3389/fonc.2023.1360223

**Published:** 2024-01-24

**Authors:** Galina G. Lagos, Wafik S. El-Deiry, Liang Cheng

**Affiliations:** ^1^ Hematology-Oncology Division, Department of Medicine, Rhode Island Hospital and Brown University, Providence, RI, United States; ^2^ Legorreta Cancer Center at Brown University, The Warren Alpert Medical School, Brown University, Providence, RI, United States; ^3^ Department of Pathology and Laboratory Medicine, Brown University Warren Alpert Medical School, Providence, RI, United States

**Keywords:** precision oncology, clinical management, genitourinary cancer/malignancies, prognosis, prostate cancer, bladder cancer, kidney cancer

In the last decade we have seen an explosion in the development of new treatment options, diagnostic tools, and prognostic and predictive biomarkers for patients with genitourinary malignancies. As we learn to use these new tools, we face the challenge of appropriately identifying patients with high-risk disease that will benefit from our most aggressive treatment modalities and those with low-risk disease who are better served with more conservative management to avoid unnecessary toxicities. The articles in this Research Topic focus on approaches to stratifying patients with genitourinary malignancies in an effort to get the right treatment to the right patient at the right time.

The treatment landscape in prostate cancer is rapidly changing and we are fortunate to have a plethora of options for patients with metastatic hormone sensitive prostate cancer (mHSPC). Sigurdson et al have concisely and effectively summarized the standards of care for mHSPC as of 2020, which included androgen deprivation therapy (ADT) combined with either an androgen receptor signaling inhibitors (ARSI) or docetaxel. Since the publication of their article, two triplet therapy regimens have demonstrated superiority over ADT+docetaxel in the frontline setting. The PEACE-1 trial showed the efficacy of abiraterone/prednisone plus ADT and docetaxel (1) and the ARASENS trial established the role for darolutamide plus ADT and docetaxel (2). An unanswered question remains whether the addition of docetaxel has an added benefit over ADT+ARSI alone.

Furthermore, we have limited data to identify which patients are most appropriate for treatment intensification. In the CHAARTED trial, patients with high volume disease (HVD) were the subgroup of patients that benefited from the addition of docetaxel (3). In subgroup analysis from PEACE-1, benefit from triplet therapy was only observed for HVD patients, although in ARASENS benefit was seen across all subgroups (4). Another consideration is how we define high volume/high risk disease as we integrate PSMA-PET imaging in practice, given that prior trials used conventional imaging. Prospective trials using novel imaging modalities and biomarkers will be needed to better define the most appropriate frontline treatment for patients with mHSPC (Rosinha et al.).

In the metastatic castrate resistant prostate cancer (mCRPC) space, individualizing treatment based on molecular biomarkers has advanced patient care. Pathogenic variants in homologous recombination repair genes are identified in 20-30% of patients with mCRPC. These mutations confer sensitivity to PARP inhibitors. Olaparib was the first approved PARPi in mCRPC after progression on a prior ARSI based on the PROfound trial (5). Although the FDA granted approval for olaparib for multiple HRR gene variants, the benefit was most pronounced in patients with *BRCA2* mutations, which has subsequently been seen across multiple PARPi trials. In the last year, on the basis of the PROpel, MAGNITUDE, and TALAPRO-2 studies, olaparib+abiraterone/prednisone, niraparib +abiraterone/prednisone, and talazoparib+enzalutamide, respectively, were all approved as first line regimens for mCRPC (6–8). In this setting, olaparib and niraparib were only approved in *BRCA* variants, although talazoparib has a broader approval for HRR mutations. Interestingly, in the TALAPRO-2 and PROpel studies, benefit was also seen in those without HRR mutations, although this benefit was less pronounced.

Another subgroup of patients where a biomarker-based approach has been valuable are the roughly 3% of prostate cancer patients with a microsatellite instability-high/mismatch repair deficient (MSI-H/MMRd) phenotype (9). In all-comers, outcomes with immune checkpoint inhibitors (ICI) in mCRPC have been disappointing across multiple trials including single agent and combination approaches. In a small series of patients with MSI-H/MMRd the response rate to checkpoint inhibitors are in the 45-53% range (9, 10). Based on the available data, pembrolizumab is now approved for this subset of patients, as well as those with high-TMB.

The question of treatment escalation or de-escalation is also relevant in the approach to earlier stages of prostate cancer. Champion et al describe the use of PSMA-PET in tailoring radiation approaches in post-prostatectomy patients. A third to one half of patients who undergo prostatectomy develop biochemical relapse. PSMA-PET scans have quickly become the preferred imaging modality to identify prostate cancer relapse in these patients. Given the novelty of this technology, most of the data regarding dose adjustment based on imaging findings is retrospective in nature. Based on the available data, PSMA-PET alone should not be used to guide radiation strategies but prospective studies are underway to help address this question (i.e. NCT04557501, NCT05067660, NCT05022914, NCT04794777). For the future, other biomarkers such as genomic classifiers and artificial intelligence-based tools may be incorporated to personalize treatment decisions.

Burke addresses the debate over our classification of low grade Gleason score 6 prostate cancers. Although some have argued to reclassify Gleason score 6 tumors as noncancer given their low mortality risk, Dr. Burke asserts that a severity prognostic score should not be used to deny the existence of a diagnosis. Despite a shared histology, not all Gleason 6 tumors are equal and over 50% of men with low-risk prostate cancer eventually receive cancer directed treatment. This speaks to the need for more granular prognostic biomarkers that can help provide reassurance to patients that will likely never need treatment and to prepare patients who may benefit from treatment down the line.

Beyond prostate cancer, there has been significant investment in developing better prognostic and predictive biomarkers to guide bladder cancer treatment. In muscle invasive bladder cancer (MIBC), the standard treatment for eligible patients is neoadjuvant chemotherapy followed by radical cystectomy. Given the morbidity and mortality risks, organ-sparing approaches are an attractive alternative. Trimodality therapy (TMT) using maximal transurethral resection of bladder tumor (TURBT) followed by chemotherapy with radiation is an option for patients who decline or are unfit for cystectomy. In current practice we use clinical factors including patient and tumor characteristics to identify candidates for TMT. For carefully selected patients, outcomes may be similar between TMT and radical cystectomy (11). Currently there are no validated biomarkers to select patients for TMT but areas of active investigation include molecular subtypes based on transcriptomic profiles, gene expression signatures of immune activation, and DNA damage response mutations (12). Ferro et al. describe pre-treatment lymphocyte-to-monocyte ratio (LMR) as another promising biomarker to identify patients at highest risk for progression which may guide therapy decisions.

It has also been observed that a subset of patients achieves long term bladder intact disease-free survival after TURBT plus systemic (13, 14). There are several completed trials and some underway to evaluate the role of TURBT plus systemic therapy as a definitive treatment option (15–17). Some of these trials use biomarkers, including the presence of functionally deleterious DNA damage response gene alterations, which are associated with improved response to cisplatin-based therapies, to select appropriate candidates for observation after completion of chemotherapy. They also use stringent criteria to select patients who may avoid cystectomy, including a negative restaging TURBT, negative urine cytology, and no evidence of disease on MRI. Translational analyses from these studies will help define genomic, immunologic, and radiologic biomarkers to optimize patient selection for treatment approaches.

The newly evolving standard of care systemic therapy from metastatic bladder cancer is also likely to make its way in the earlier disease stages and broaden treatment options. As of this year the combination of enfortumab vedotin, an antibody drug conjugate targeting Nectin-4, with pembrolizumab, a PD-1 inhibitor, has been approved as an alternative to platinum based chemotherapy, which was been the frontline standard for decades. The phase III EV-302 trial demonstrated the superiority of this combination, which nearly doubled median OS and PFS relative to platinum based chemotherapy. Importantly, this effect was observed across pre-defined subgroups including cisplatin eligibility and PD-L1 expression (18). EV-304 is an ongoing trial exploring this combination in the neoadjuvant setting for muscle invasive disease with results eagerly awaited.

As therapeutic options for patients with genitourinary malignancies continue to expand, we must continue our efforts to tailor the right treatments for individual patients. Current research endeavors highlight evolving landscape of clinical management of genitourinary malignancies and the innovative approaches in development that will continue to push the field forward in coming years.

## Author contributions

GL: Writing – original draft, Writing – review & editing. WE-D: Writing – original draft, Writing – review & editing. LC: Writing – original draft, Writing – review & editing.
